# Dyeing of Polyester with 4-Fluorosulfonylphenylazo-5-pyrazolone Disperse Dyes and Application of Environment-Friendly Aftertreatment for Their High Color Fastness

**DOI:** 10.3390/ma12244209

**Published:** 2019-12-14

**Authors:** Sanghyun Yoon, Byunghun Choi, Md Morshedur Rahman, Santosh Kumar, Shekh Md Mamun Kabir, Joonseok Koh

**Affiliations:** 1 Department of Organic and Nano System Engineering, Konkuk University, 120 Neungdong-ro, Gwangjin-gu, Seoul 05029, Korea; sucre92@naver.com (S.Y.); knight8603@nate.com (B.C.); morshed133@gmail.com (M.M.R.); 2 Division of Chemical Engineering, Konkuk University, 120 Neungdong-ro, Gwangjin-gu, Seoul 05029, Korea; santoshics@gmail.com; 3 Department of Wet Processing Engineering, Bangladesh University of Textiles, Dhaka 1208, Bangladesh; mamunkabir.butex@gmail.com

**Keywords:** 4-arylazo-5-pyrazolone dyes, fluorosulfonyl group, polyester, build-up properties, fastness properties, alkali clearing

## Abstract

Dyeing and fastness properties of a series of 4-fluorosulfonylphenylazo-5-pyrazolone dyes on polyester were investigated in this study. The 4-nitrophenylazo-5-pyrazolone dyes were also synthesized to compare their dyeing and fastness properties on polyester with those of fluorosulfonyl-substituted analogues. The substantivity of 4-arylazo-5-pyrazolone derivatives containing a *p*-fluorosulfonyl group in the diazo component was lower than that of their nitro analogues which have a higher extinction coefficient and higher affinity because of the polar nitro group. They showed relatively hypsochromic color and lower chroma on polyester compared with their nitro analogues because of the relatively weaker electron-accepting power of the fluorosulfonyl group compared to the nitro group. Disperse dyeing of polyester with 4-fluorosulfonylphenylazo-5-pyrazolone disperse dyes achieved high color fastness and reduces the adverse environmental impact of the dyeing process by providing the option of performing alkali clearing instead of reductive clearing, which has high biological oxygen demand when discharged into the dyeing effluent and generates carcinogenic aromatic amines.

## 1. Introduction

**Scheme 1:**
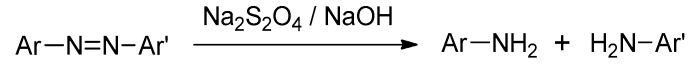
Reductive hydrolysis of conventional azo disperse dyes during reduction clearing (Ar, Ar’ = substituted aryl).

Polyester, specifically polyethylene terephthalate (PET), is the most widely used material in the textiles industry because of its excellent physical properties, processability, lower price, etc., [1]. In a typical textile dyeing process, PET is dyed with disperse dyes in a weakly acidic aqueous dyebath at 130 °C, and some particulate disperse dye molecules may occlude onto the fiber surface after the completion of dyeing because of the limited water-solubility. If the dye on the surface is not removed, then the overall color fastness of dyed fabrics can be undermined, especially in heavy depth dyeing. Therefore, to achieve acceptable color fastness properties in disperse dyeing of polyester, excess dye on the fiber surface is usually cleared by reductive treatment, where the dyed fabric is treated in a bath made up of a reducing agent (i.e., sodium hydrosulfite) and a strong alkali (i.e., sodium hydroxide) [2]. However, reduction clearing as aftertreatment has an adverse environmental impact in addition to its expense because of the highly alkaline conditions, discharge of high levels of sulfur with the wastewater, and substantial amounts of water used. There is also concern that wastewater from reduction clearing contains carcinogenic or mutagenic aromatic amines from the reduction of azo dyes (Scheme 1) [3,4,5,6]. To solve these environmental problems in the textile dyeing industry, alkali-dischargeable azo disperse dyes have been proposed, which eliminate the need for a strong alkali and toxic reducing agent, thus reducing the cost of effluent treatment and the negative environmental impact [7,8,9,10].

**Scheme 2:**
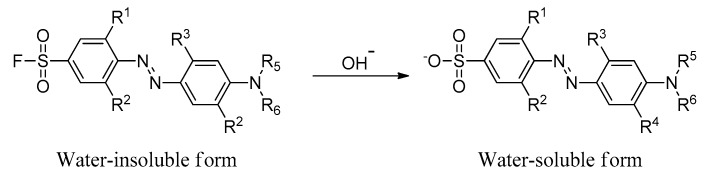
Hydrolysis of 4-amino-4′-fluorosulfonylazobenzene dyes under alkaline condition (R^1^, R^2^, R^3^, R^4^, R^5^, and R^6^ = substituents).

Some novel alkali-clearable azo disperse dyes incorporating a fluorosulfonyl group were investigated in our previous studies [11,12,13,14,15]. Azo disperse dyes containing a fluorosulfonyl group hydrolyzed under mild alkaline conditions to water-soluble dyes containing a sulfonate group without scission of the azo linkage and could be easily washed-off (Scheme 2). The hydrolysis of fluorosulfonyl group in alkaline solution followed pseudo first-order kinetics, and the temperature showed a much greater effect on the hydrolysis rate than pH of the solution [9,12]. 4-(N,N-diethylamino)-4′-fluorosulfonylazobenzene dyes covered colors from red to greenish blue (469–620 nm) in ethanol solution [9]. Recently, we synthesized some pyrazolone-based heterocyclic yellow azo disperse dyes containing a fluorosulfonyl group, and the substituent effects on their spectral and thermal properties were investigated [15]. Heterocyclic azo disperse dyes are known to achieve brighter dyeing and better tinctorial strength than their benzenoid counterparts [16,17,18]. Pyridones and pyrazolones have been shown to be important dye intermediates especially for coupling components of yellow to orange azo dyes in the textile dyeing industry [19]. However, since the introduction of 4-fluorosulfonylphenylazo-5-pyrazolone disperse dyes [15], very little information is available in their application to polyester. Therefore, it is worth comparing their dyeing properties on PET with conventional azo disperse dyes, i.e., 4-nitrophenylazo-5-pyrazolone disperse dyes.

In this study, the dyeing and fastness properties of a series of 4-fluorosulfonylphenylazo-3-methyl-5-pyrazolone azo dyes on PET were investigated. To confirm the feasibility of alkali clearing as an alternative aftertreatment to noxious reduction clearing in the PET dyeing process, the hydrolysis behaviors of the dyes at alkali-clearing and reduction-clearing conditions were compared using HPLC (high-pressure liquid chromatography).

## 2. Materials and Methods

### 2.1. Materials and Reagents

PET woven fabrics (70 ± 5 g/m^2^; plain weave, warp, and weft each 75-denier/24-filament, KS K 0905) were used for disperse dyeing. All chemicals used in this study were of laboratory reagent grade. Diwatex (sodium lignosulfonate, Borregaard Lignotech, Sarpsborg, Norway) was used as the dispersing agent for milling, along with Sandozin NIE (alkyl phenol ethoxylate, Clariant, Muttenz, Switzerland) as the wetting agent. Lyocol RDN liquid (polyaryl ether sulfate, Clariant, Muttenz, Switzerland) was used as the dispersing agent in PET dyeing.

### 2.2. Dye Synthesis and Preparation of Dye Dispersions

4-Arylazo-3-methyl-5-pyrazolone dyes **1** and **2** (Table 1) were synthesized as described in a previous study [15]. Numerous spectroscopic investigations showed that the tautomeric equilibrium of phenylazopyrazolone dyes favors the hydrazone form over the azo form (Table 1) in the polar solvents and solid state [20,21,22]. Mixtures of wetting agent (1 drop), dispersing agent (40% on weight of dye), and 1.0 g of the synthesized dye were milled with glass beads for 24 h in 100 mL of water buffered at pH 4.5 for the preparation of 1.0 w/v% of dye dispersion.

### 2.3. Hydrolysis Analysis at Alkali-Clearing and Reduction-Clearing Conditions

Hydrolysis (%) of dyes **1b** and **2b** at alkali clearing (NaOH 1.0 g/L at 80 °C for 20 min) and reduction clearing (NaOH 2.0 g/L and Na_2_S_2_O_4_ 2.0 g/L at 80 °C for 20 min) conditions was measured by HPLC to compare their washing efficiencies, which determine the color fastness of dyed PET. The dyebath was prepared by dispersing 1.0 mL of dye dispersion (1.0 w/v%) in 49 mL of distilled water. The temperature was raised from room temperature to 80 °C at a rate of 2 °C/min; then, the temperature was kept stable for 20 min using a laboratory infrared dyeing machine (DLS-6000, Daelim Starlet Co., Ltd., Siheung, Korea). When clearing was completed, aliquots were withdrawn from the dye solution and immediately neutralized to pH 4.5 with appropriate amounts of dilute hydrochloric acid solution, and then followed by cooling to prevent further hydrolysis. A specific amount of acetonitrile was added to each of the neutralized samples to adjust the eluent composition to be acetonitrile(80)/water(20) (v/v) for HPLC analysis. HPLC analysis was carried out at room temperature using the ACHE 9000 (Younglin Instrument Co., Ltd., Anyang, South Korea) with a C18 reversed-phase column (Waters Korea, SunFire, Seoul, South Korea, 100 Å, 5 μm spherical silica, 4.6 mm × 150 mm). An acetonitrile-deionized water mixture (80:20 v/v) was used as the mobile phase and its flow rate was set at 1.0 m/min. When the HPLC system was stabilized, 10 μL of the samples were injected and detected at 449 nm.

The UV-visible spectra of dyes **1b** and **2b** treated by reduction clearing and alkali clearing were measured every 10 min while the temperature of the dye solution was raised from 20 to 80 °C using a laboratory dyeing machine coupled with a UV-visible spectrophotometer (Dye Max-L, Dyetex Eng. Co. Ltd, Seongnam, Korea).

### 2.4. Dyeing

PET fabrics were dyed in a laboratory infrared dyeing machine. A total of 40 mL of dyebath containing a certain amount of the above dye dispersion and 1.0 mL/L of dispersing agent were prepared and their pH was adjusted to pH 4.5 with acetic acid. A PET woven fabric (2.0 g) was then immersed in the prepared dyebath and the temperature was raised to 130 °C at a rate of 2 °C/min, followed by keeping the temperature stable for 60 min using a laboratory dyeing machine.

The color properties (CIE L*, a*, b*, C*, and *h_a_*_b_) of the dyed PET fabrics were measured using an X-Rite 8000 Series (X-Rite, Inc., Grand Rapids, MI, USA) at a specific color measurement condition (10° standard observer, standard light D65 and specular component included).

The *f_k_* values of dyed fabrics at various dye concentrations (0.5, 1.0, 2.0, and 4.0% on mass of fabric) were measured in order to compare the build-up properties of dyes **1** and **2** on PET. The color strength value, *f_k_*, is taken as the sum of the weighted K/S values in the visible region of the spectrum by using Equation (1) [23].

(1) $$f_{k}\;=\;\sum_{\lambda =400}^{700}{\left(K/S\right)}_{\lambda }({\bar{x}}_{10,\lambda }+{\bar{y}}_{10,\lambda }+{\bar{z}}_{10,\lambda })$$

where ${\bar{x}}_{10,\lambda }$, ${\bar{y}}_{10,\lambda }$, and ${\bar{z}}_{10,\lambda }$ are the color matching functions for the 10° standard observer at each wavelength (ISO 7724/1-1984).

### 2.5. Color Fastness Test

To evaluate the clearing efficiency of the synthesized dyes, samples that received three different aftertreatments, i.e., reduction-cleared (RC), alkali-cleared (AC), and non-cleared (NC), were prepared after the dyeing. The three different samples were heat-set at 180 °C for 30 s prior to the color fastness test.

The dye concentration of 4.0%omf (on mass of fabric) was chosen for the color fastness tests. The reason is because the dyed PET, especially at heavy depth dyeing, must usually be reduction cleared to obtain enhanced color fastness. The color fastness test was carried out according to the international standards: ISO 105-C06/B1M for fastness to washing, ISO 105-X12 for fastness to rubbing, and ISO 105-E04 for fastness to perspiration. Staining of adjacent multifiber (Multifibre DW, BS EN ISO 105-F10) and changes in color shade were assessed using gray scales (ISO 105-A02 and A03).

## 3. Results

### 3.1. Hydrolysis Analysis at Alkali-Clearing and Reduction-Clearing Conditions

It is generally accepted that sulfonyl halides are hydrolyzed to sulfonic acid by a nucleophilic bimolecular substitution (S_N_2) mechanism under alkaline condition [24,25]. Therefore, it is presumed that azo disperse dyes incorporating fluorosulfonyl group can be hydrolyzed under alkaline conditions to high-washable dyes containing a sulfonate group without breakage of the azo linkage, and they can be easily washed off (Scheme 2). Indeed, in the previous study, HPLC confirmed that the observed retention time of the hydrolyzed dye from the parent dye containing a fluorosulfonyl group under alkaline conditions was exactly the same as that of the expected hydrolyzed form, 4-(4-diethylamino-2-methyl-phenylazo)benzene sulfonic acid. Moreover, there were no traces of any other side reaction products (i.e., potentially toxic primary aromatic amines) being produced during the conventional reduction clearing [9].

**Figure 1:**
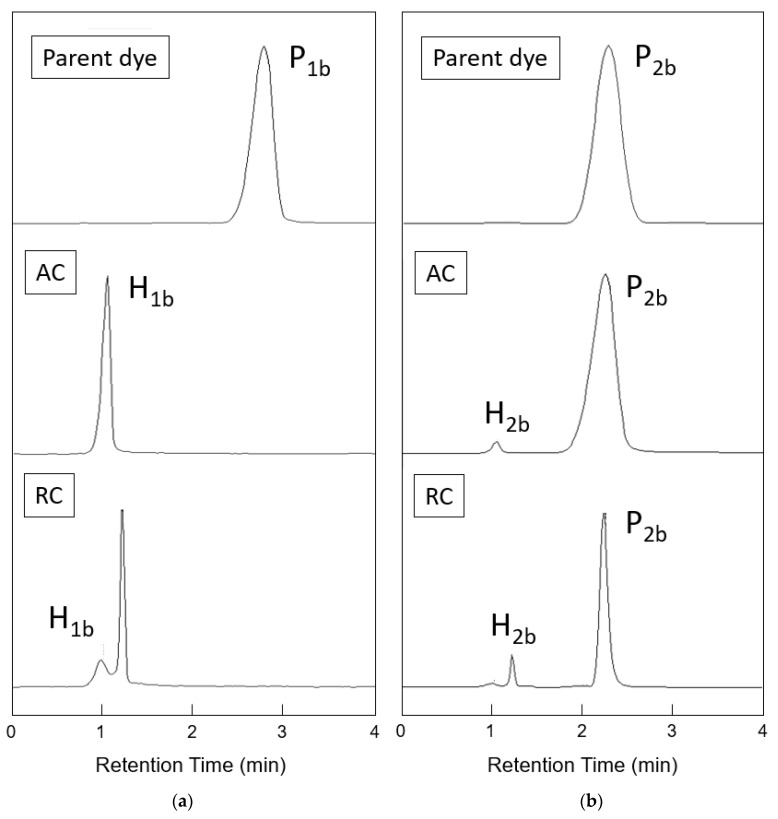
High-pressure liquid chromatography (HPLC) chromatogram of dyes **1b** and **2b** at various clearing conditions (**a**) **1b** and (**b**) **2b**.

To confirm the feasibility of alkali clearing as an alternative aftertreatment to noxious conventional reduction clearing in the PET dyeing process, the hydrolysis (%) of parent dyes **1** and **2** were compared at three different clearing conditions using HPLC (Figure 1). Dyes **1b** and **2b** exhibiting the best dyeability on PET among each of the two dye groups (dyes 1a~1c and dyes 2a~2c) were selected to compare the hydrolysis behavior at two different clearing conditions. HPLC chromatograms of dye **2b** (P_2b_ at 2.33 min in Figure 1b) showed the expected decrease in the amount of the parent dye and then an increase in the amount of the hydrolyzed form of the parent dye at both alkali-clearing and reduction-clearing conditions (H_2b_ at 1.03 min in Figure 1b). However, the amount of the hydrolyzed form was not as significant as expected, even in the RC sample. There were also traces of side reaction products (H_2b_ at 1.25 min in Figure 1b), which could be primary aromatic amines originating from the cleavage of azo linkages (Scheme 1). In the case of dye **1b**, the HPLC chromatogram of the AC sample shows the retention times of the hydrolyzed forms of the dye and parent dye at 1.10 (H_1b_ in Figure 1a) and 2.85 min (P_1b_ in Figure 1a), respectively. All the parent dyes of dye **1b** were hydrolyzed at both alkali-clearing and reduction-clearing conditions; in the case of dye **2b**, only 2.2% and 10.1% of parent dyes were hydrolyzed at alkali-clearing and reduction-clearing conditions, respectively. It is worth noting that, in the case of AC sample of dye **1b**, there were no traces of other side reaction products such as potentially toxic aromatic amines produced during reduction clearing (H_1b_ and H_2b_ at retention time 1.25 min in Figure 1a,b), as shown in the HPLC chromatogram.

**Figure 2:**
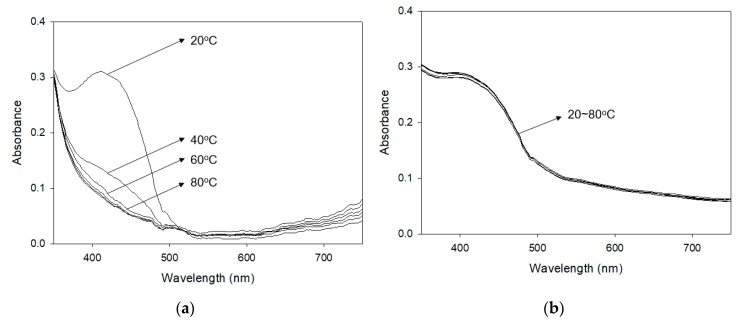
UV-visible spectra of dyes **1b** under (**a**) reduction-clearing and (**b**) alkali-clearing conditions while the temperatures of dye solutions were raised from 20 to 80 °C.

Figure 2 shows the changes in the absorption spectra of dyes **1b** resulting from reduction clearing and alkali clearing, respectively. In the case of reduction clearing, the absorbance at λ_max_ tends to decrease over time, since the azo bond of the dye was cleaved by the action of the reducing agent and the dye became colorless. However, in the case of alkali clearing, the absorption spectrum did not show any significant changes in absorbance during clearing, since the azo linkage of the dye was not susceptible to hydrolysis under relatively mild alkaline conditions.

**Scheme 3:**
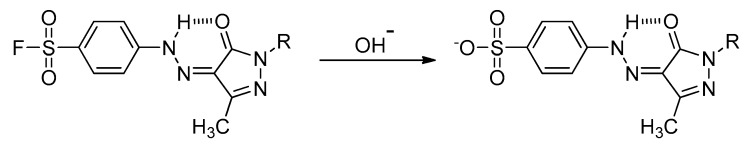
Alkaline hydrolysis of 4-fluorosulfonylphenylazo-3-methyl-5-pyrazolone disperse dyes (R = H, phenyl, *p*-chlorophenyl, etc.).

All the results shown in Figure 1 and Figure 2 indicate that the fluorosulfonyl group in the structure of dye **1b** was converted into a high-washable form without breakage of the azo group by hydrolysis under mild alkaline conditions (Scheme 3).

### 3.2. Dyeing Properties on PET

#### 3.2.1. Build-Up Properties on PET

**Figure 3:**
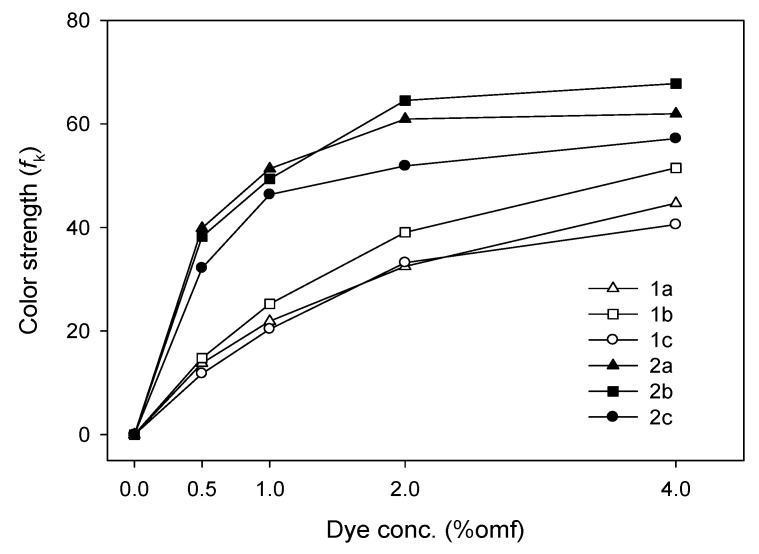
Build-up properties of dyes **1** and **2** on polyethylene terephthalate (PET).

Figure 3 shows the increase in color strength (*f_k_*) of dyed PET fabrics with the applied dye concentration (%omf). Although the build-up properties of the synthesized arylazo pyrazolones dyes were relatively poor compared to 4-aminoazobenzene dyes because of the lower tinctorial strength and substantivity to PET [26], they showed a reasonable level of build-up properties on PET, and saturation was reached with the application of approximately 4.0%omf. Arylazo pyrazolone dyes containing a fluorosulfonyl group (dye **1**) exhibited slower build-up and lower color yield at saturation points compared with nitro analogues. Thus, in dyeing PET fiber, the substantivity of arylazo pyrazolone derivatives containing a *p*-fluorosulfonyl group in the diazo component was shown to be lower than that of their nitro analogues, which have a higher extinction coefficient and higher affinity because of the polar nitro group. For example, dye **1b** yielded lower depth (*f_k_* 25.24) than its nitro analogue, dye **2b** (*f_k_* 49.39) at 1.0%omf dyeing. Similarly, dyes **1a** and **1c** produced lower *f_k_* values of 21.93 and 20.40, respectively, compared with the nitro analogues **2a** (*f_k_* 51.38) and **2c** (*f_k_* 46.38). Indeed, many commercial azo disperse dyes for PET dyeing contain the *p*-nitro group in the diazo component to enhance their build-up property.

#### 3.2.2. Color Properties on PET

The color properties of the dyed PET with dyes **1** and **2** are shown in terms of the CIELAB system (Table 2). The colors of dyes **1** and **2** on PET range from yellow to orange, which is different from that of 4-(*N*,*N*-diethylamino)-azobenzene dyes covering reddish-orange to dark violet [9]. In the previous study, the chroma (C*) values of the dyed PET with 1.0%omf of the analogous dyes of dyes **1a** and **2a** containing N,N-diethylamino group in place of pyrazolone group were 73.00 and 52.19, respectively [26]. Table 2 shows that chroma values of dyes **1a** (C* = 85.68) and **2a** (C* = 94.09) exhibited higher values than those of their 4-aminoazobenzene-based analogues. Indeed, the heterocyclic azo disperse dyes are characterized by brighter dyeing than their benzenoid counterparts [16,17,18].

**Figure 4:**
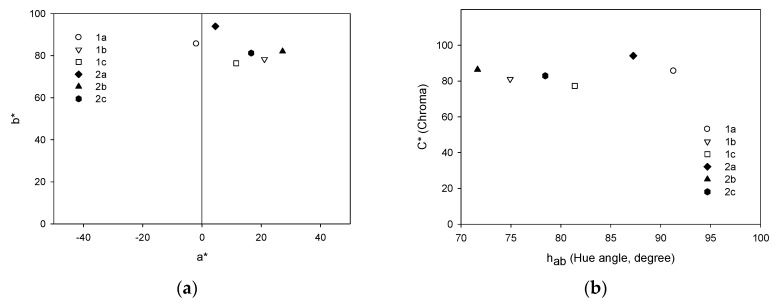
Color properties of dyed PET with dyes **1** and **2**: (**a**) CIELAB a* vs. b* and (**b**) CIELAB hue angle vs. chroma.

**Figure 5:**
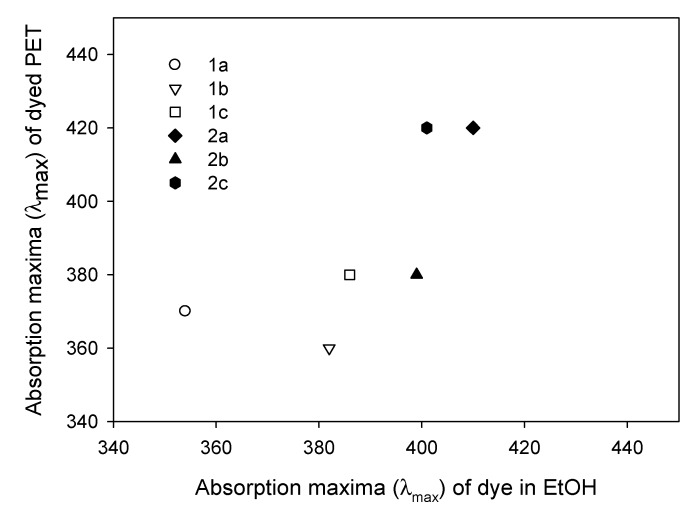
Correlation between absorption maxima of dyes in ethanol (EtOH) solution and dyed PET.

Nitro-substituted dyes (**2a**, **2b**, and **2c**) show relatively higher chroma (C*) than fluorosulfonyl-substituted dyes (**1a**, **1b**, and **1c**) (Figure 4). As expected, the fluorosulfonyl-substituted dyes were slightly hypsochromic on PET compared with their nitro analogues because of the relatively lower electron-accepting power of the fluorosulfonyl group (λ_max_ in Table 1 and h_ab_ in Table 2) [27]. These trends in color properties of the dyed PET are consistent with the spectral properties of the dyes in ethanol solution, as shown in Figure 5.

### 3.3. Color Fastness Properties

#### 3.3.1. Fastness to Washing

**Figure 6:**
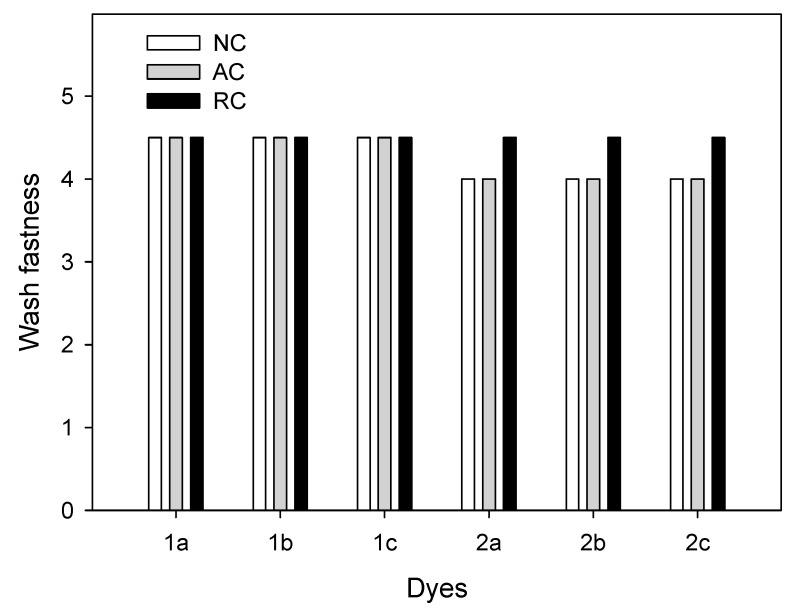
Color fastness to washing (staining on nylon) of dyed PET with dyes **1** and **2**.

The strips of nylon and diacetate in adjacent multifiber fabrics are prone to staining during the wash fastness test of the disperse-dyed PET because of their relatively hydrophobic characteristics. Wash fastness ratings of the dyed PET in terms of staining on nylon and diacetate are shown in Table 3 and Figure 6. In the cases of dyes **1a**, **1b**, and **1c** containing the fluorosulfonyl group, not only the RC samples but also the AC and even NC samples exhibited similar high fastness, presumably because the fluorosulfonyl group of dyes **1** was hydrolyzed to a water-soluble group under alkaline conditions during the aftertreatment and/or wash fastness test procedure, as investigated in previous studies [9,12]. The dye residues were therefore more readily washed-off and exhibited low staining on adjacent multifiber fabric in the wash fastness test. These results are consistent with the hydrolysis analysis results shown in Figure 1 and Figure 2.

PET dyeing was also carried out for comparison using 4-nitrophenylazo-3-methyl-5-pyrazolone dyes (dyes **2a**, **2b**, and **2c**) which were not expected to be alkali-clearable since the nitro group is not susceptible to hydrolysis under mild alkaline conditions. Although the fastness to washing of RC samples ranged from very good to excellent (ratings of 4–5), NC and AC samples showed some staining (rating of 4) on the strips of nylon in adjacent multifiber fabrics (Table 3). An analogous comparison between the 4-fluorosulfonylphenylazo-3-methyl-5-pyrazolone dyes and their nitro analogues revealed a gap in alkali-clearability, exemplified by the staining of nylon (Figure 6). The nitro analogues exhibited lower fastness by as much as 0.5 to 1 grading units in the case of AC samples. These results show that alkaline clearing of dyed PET with 4-arylazo-5-pyrazolone dyes incorporating a fluorosulfonyl group produce excellent wash fastness, irrespective of the clearing methods applied.

#### 3.3.2. Fastness to Perspiration

**Figure 7:**
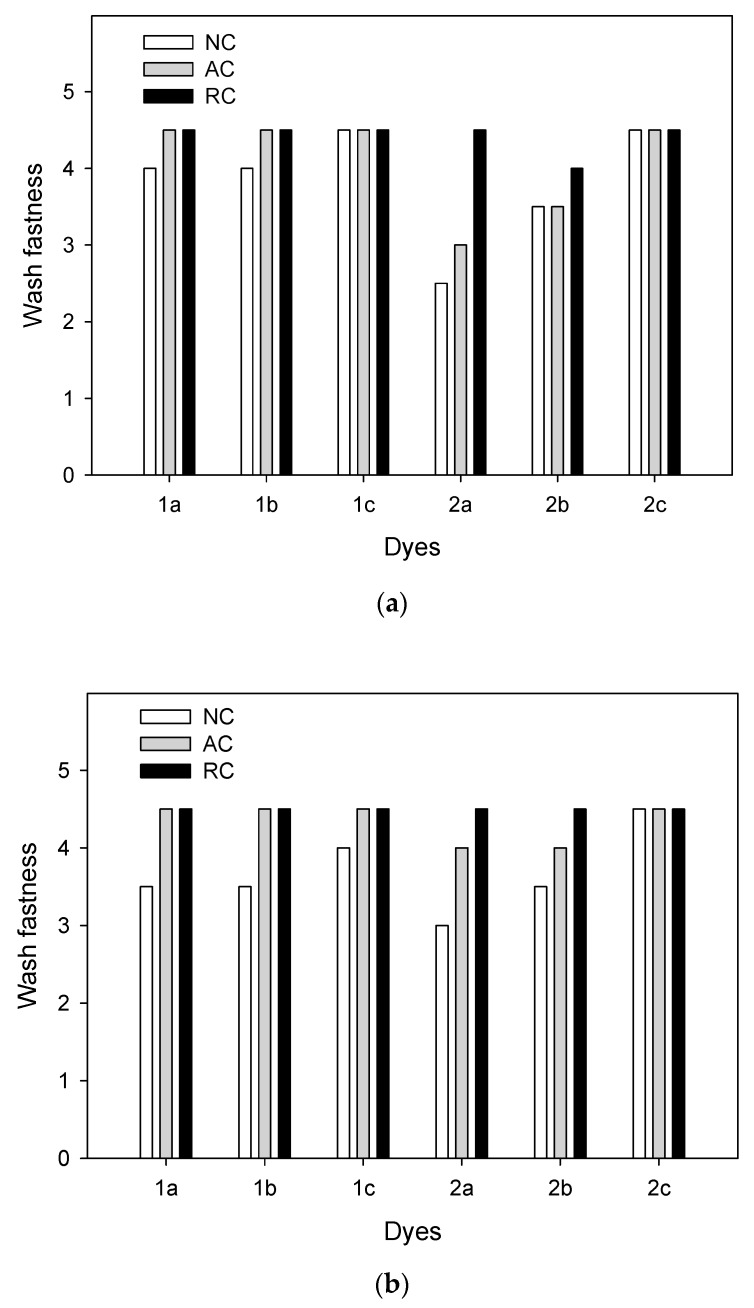
Fastness to perspiration (staining on nylon) of dyed PET with dyes **1** and **2**: (**a**) acid perspiration and (**b**) alkali perspiration.

4-Fluorosulfonylphenylazo-3-methyl-5-pyrazolone dyes (**1a**, **1b**, and **1c**) exhibited lower staining in perspiration fastness tests compared to the nitro-substituted analogues (**2a**, **2b**, and **2c**), even without any clearing treatment; in the case of NC samples, perspiration fastness of dyes **1** was higher than that of dyes **2** by as much as 0.5 to 1.5 grading units (Table 4 and Figure 7).

In the case of 4-nitrophenylazo-3-methyl-5-pyrazolone dyes (**2a**, **2b**, and **2c**), although NC samples caused some staining (ratings of 2–3 to 5), the fastness to perspiration of RC samples ranged from good to excellent (ratings 4 to 5). Alkali clearing also improved fastness levels (ratings of 3 to 5), but to a lesser extent compared with the reduction clearing. On the other hand, in the case of fluorosulfonyl analogues, not only RC samples but also AC samples did not stain nylon at all (rating of 5). Again, these results claim that 4-fluorosulfonylphenylazo-3-methyl-5-pyrazolone dyes have excellent perspiration fastness properties on PET, irrespective of the clearing method applied.

#### 3.3.3. Fastness to Rubbing

The fastness to rubbing of dyed PET with dyes **1** and **2** ranged from very good to excellent regardless of whether reduction or alkali clearing was applied, except for a few cases (Table 5). Dyes **1** exhibited slightly higher grades than dyes **2** in some cases, especially in terms of wet rubbing fastness.

## 4. Conclusions

The dyeing and fastness properties of 4-fluorosulfonylphenylazo-3-methyl-5-pyrazolone dyes and their nitro analogues on PET were investigated in a comparative manner in this study. To confirm the feasibility of alkali clearing as an alternative aftertreatment to conventional noxious reduction clearing in PET dyeing, the hydrolysis (%) of parent dyes at alkali-clearing and reduction-clearing conditions was compared using HPLC.

In disperse dyeing of PET, the substantivity of 4-arylazo-5-pyrazolone derivatives containing a *p*-fluorosulfonyl group in the diazo component was lower than that of their nitro analogues which have a higher extinction coefficient and higher affinity because of the polar nitro group. They showed relatively hypsochromic color and lower chroma on polyester compared to their nitro analogues because of the relatively weaker electron-accepting power of the fluorosulfonyl group compared to the nitro group. The fastness properties of 4-arylazo-5-pyrazolone disperse dyes containing a fluorosulfonyl group on PET fabrics were excellent. Not only the reduction-cleared fabrics but also the alkali-cleared and even non-cleared fabrics showed good color fastness. These results are attributed to the outstanding wash-off properties resulting from the alkali-clearability imparted by the alkaline hydrolysis of fluorosulfonyl residues. These findings were also supported by the hydrolysis analysis results using an HPLC and UV-visible spectrophotometer. It is thus expected that the application of 4-arylazo-5-pyrazolone disperse dyes containing a fluorosulfonyl group on PET will be able to reduce the adverse environmental impact of the dyeing process by providing the option of performing alkali clearing instead of reductive clearing, which generates carcinogenic aromatic amines and has high BOD (biological oxygen demand) when released in the dyeing effluent.

## Figures and Tables

**Table 1:**
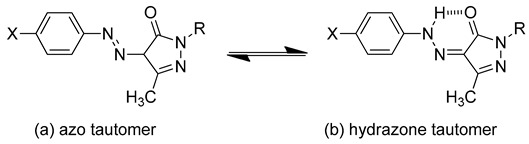
4-Arylazo-3-methyl-5-pyrazolone dyes **1** and **2** [15].

Dye	X	R	Mol. Weight	Melting Point	$\lambda_{max}^{EtOH}$	$\varepsilon_{max}^{EtOH}$	Appearance
**1a**	FO_2_	H	284.27	230.5 °C	354 nm	18,000	Yellow solid
**1b**	FO_2_	Ph	360.36	163.2 °C	382 nm	26,000	Orange-yellow solid
**1c**	FO_2_	Ph-*p*Cl	394.81	205.0 °C	386 nm	26,000	Orange-yellow solid
**2a**	NO_2_	H	247.21	269.6 °C	410 nm	23,000	Yellow solid
**2b**	NO_2_	Ph	323.31	199.8 °C	399 nm	31,000	Orange-yellow solid
**2c**	NO_2_	Ph-*p*Cl	357.75	243.4 °C	401 nm	29,000	Orange-yellow solid

**Table 2:** Colorimetric data and visual appearance of the dyed PET fabrics with dyes **1** and **2** (1.0%omf).

Dye	L*	a*	b*	C*	*h_ab_*	Color on PET
**1a**	87.57	−1.91	85.66	85.68	91.28	Vivid yellow
**1b**	79.31	21.08	78.27	81.06	74.93	Strong orange-yellow
**1c**	83.13	11.56	76.36	77.23	81.39	Brilliant orange-yellow
**2a**	85.32	4.52	93.98	94.09	87.25	Vivid yellow
**2b**	76.29	27.19	82.04	86.43	71.66	Vivid orange
**2c**	79.98	16.61	81.23	82.91	78.44	Strong orange-yellow

**Table 3:** Fastness to washing of dyed PET with dyes **1** and **2** (ISO 105 C06 B1M).

Dye	NC		AC		RC	
	D^a^	N^b^	D	N	D	N
**1a**	5	4–5	5	4–5	5	4–5
**1b**	5	4–5	5	4–5	5	4–5
**1c**	5	4–5	5	4–5	5	4–5
**2a**	4	4	4	4	5	4–5
**2b**	4–5	4	4–5	4	4/5	4–5
**2c**	4–5	4	4–5	4	4/5	4–5

D^a^: Diacetate, N^b^: Nylon.

**Table 4:** Fastness to perspiration of dyed PET with dyes **1** and **2** (ISO 105 E04).

Dye	Alkali					Acid						
	NC		AC		RC		NC		AC		RC	
	D	N	D	N	D	N	D	N	D	N	D	N
**1a**	4–5	3–4	4–5	4–5	5	4–5	4–5	4	4–5	4–5	5	4–5
**1b**	4–5	3–4	4–5	4–5	5	4–5	4–5	4	4–5	4–5	5	4–5
**1c**	4–5	4	4–5	4–5	5	4–5	5	4–5	4–5	4–5	5	4–5
**2a**	2	3	4	4	4–5	4–5	3	2–3	4	3	4–5	4–5
**2b**	4	3–4	4–5	4	4–5	4–5	4–5	3–4	4–5	3–4	4–5	4
**2c**	4–5	4–5	4–5	4–5	4–5	5	4–5	4–5	4–5	4–5	5	5

**Table 5:** Fastness to rubbing of dyed PET with dyes **1** and **2** (ISO 105 X12).

Dye	NC		AC		RC	
	Dry	Wet	Dry	Wet	Dry	Wet
**1a**	4–5	3–4	4–5	4–5	4–5	4–5
**1b**	4–5	4–5	4–5	4–5	4–5	4–5
**1c**	4–5	4–5	4–5	4–5	4–5	4–5
**2a**	4–5	3–4	4–5	3–4	4–5	4
**2b**	4	4	4–5	4–5	4–5	4–5
**2c**	4–5	4–5	4–5	4–5	4–5	4–5
